# Supplementary material for: Expression and Characterization of a Novel Cold-Adapted Chitosanase from Marine Renibacterium sp. Suitable for Chitooligosaccharides Preparation
Source: Mar Drugs. 2021 Oct 21;19(11):596. doi: 10.3390/md19110596 (PMC8620120; doi:10.3390/md19110596)
Supplement: Supplementary file 1 [file marinedrugs-19-00596-s001.zip › marinedrugs-1397599-supplementary.pdf]

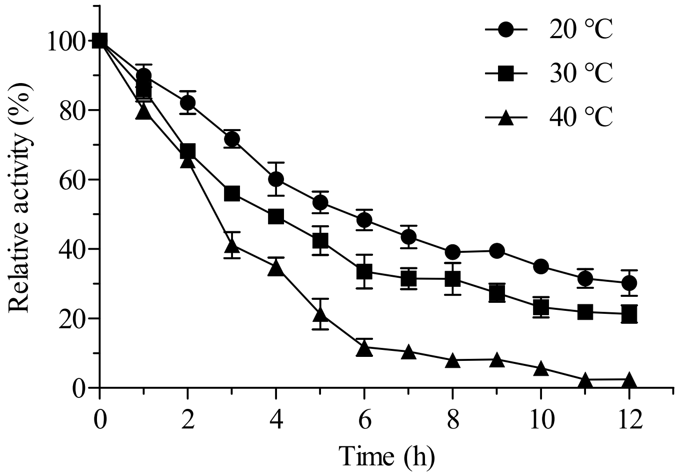

Figure S1. Thermal stability of CsnY within 12 h. The residual activities of CsnY were measured at various time intervals during incubation at certain temperatures (20 °C, 30 °C, and 40 °C).

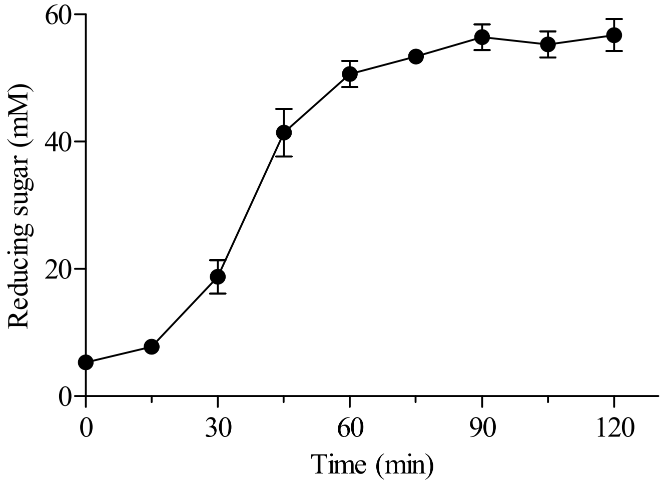

Figure S2. Catalytic hydrolysis of chitosan by CsnY. The amounts of reducing sugar generated during hydrolysis were monitored using 3,5-dinitrosalicylic acid (DNS) method.
